# Sex-Specific Association of Toll-like Receptor 8 Polymorphisms with COVID-19 Case Status in a Korean Population

**DOI:** 10.3390/life16071167

**Published:** 2026-07-14

**Authors:** Mohammed Zayed, Yong-Chan Kim, Chang-Seop Lee, Byung-Hoon Jeong

**Affiliations:** 1 Korea Zoonosis Research Institute, Jeonbuk National University, Iksan 54531, Republic of Korea; mzayed2@vet.svu.edu.eg; 2 Department of Bioactive Material Sciences, Jeonbuk National University, Jeonju 54896, Republic of Korea; 3 Department of Surgery, College of Veterinary Medicine, Qena University, Qena 83523, Egypt; 4 School of Life Sciences and Biotechnology, Gyeongkuk National University, Andong 36729, Republic of Korea; kych@anu.ac.kr; 5 Department of Internal Medicine, Research Institute of Clinical Medicine, Jeonbuk National University, Jeonju 54907, Republic of Korea; 6 Biomedical Research Institute, Jeonbuk National University Hospital, Jeonju 54907, Republic of Korea

**Keywords:** coronavirus disease 2019, TLRs, association study, SNPs, genetics, variants, innate immunity

## Abstract

Severe acute respiratory syndrome coronavirus 2 (SARS-CoV-2) is the causative pathogen of coronavirus disease 2019 (COVID-19). Toll-like receptor 8 (*TLR8*), which is located on the X chromosome, plays as a key mediator of the innate immune response. Genetic variation in the form of single-nucleotide polymorphisms (SNPs) within *TLR8* has been linked to changes in the transcriptional activity of this gene. Thus, we aimed to identify *TLR8* SNPs in the proximal promoter region and investigate whether these SNPs are associated with COVID-19 case status in a Korean population. We performed amplicon sequencing to investigate the genotypes and allele frequencies of regulatory SNPs in COVID-19 patients (*n* = 191) and the control group (*n* = 173). Four polymorphic sites, rs5741883, rs186566524, rs3764879, and rs3764880, were identified within the *TLR8* proximal promoter. Given the X-linked nature of this locus, allele and genotype frequencies were computed independently by sex. Notably, the minor C allele at rs3764879 occurred at a markedly reduced rate among male patients (10%) relative to male controls (24%), corresponding to an OR of 0.35 (95% CI 0.15–0.8; *p* = 0.018; *q* = 0.036). A parallel pattern emerged for rs3764880, where the minor A allele was likewise underrepresented in male patients (9%) versus male controls (24%), yielding an OR of 0.3 (95% CI 0.12–0.7; *p* = 0.01; *q* = 0.036). By contrast, neither genotype nor allele distributions differed significantly between female patients and female controls for any of the four variants. These results indicate that the *TLR8* polymorphisms rs3764879 and rs3764880 may be associated with COVID-19 case status among Korean males, although further validation in larger, independent cohorts is required.

## 1. Introduction

Beginning in early 2020, coronavirus disease 2019 (COVID-19), driven by infection with severe acute respiratory syndrome coronavirus 2 (SARS-CoV-2), rapidly became a worldwide pandemic [1,2]. As a result, significant efforts have been initiated to develop diagnostic and therapeutic approaches targeting SARS-CoV-2 [3,4]. The host immune system (especially the innate immune system) serves as the first line of defense against SARS-CoV-2 infection by activating inflammatory pathways upon pathogen detection [5]. Among various pattern recognition receptors, members of the toll-like receptors (TLRs) family play a critical role in the innate immune response and the pathogenesis of COVID-19 [6]. Genetic polymorphisms in genes encoding TLRs have been associated with severe respiratory symptoms in COVID-19 [7]. Notably, *TLR8* has been associated with COVID-19 risk and severity [8,9]. However, the association of *TLR8* with COVID-19 has not been fully investigated in Korean populations.

TLRs distinguish pathogen-associated and damage-associated molecular patterns developed by the virus and by the host’s injured cells, respectively [10]. Due to TLR signaling, innate immune cells, including neutrophils, monocytes, and natural killer cells, migrate to the infection site to restrain the viral infection [11,12]. The TLR response ultimately stimulates the release of proinflammatory cytokines, such as tumor necrosis factor, interleukin, interferon, and chemokines, by different signaling pathways to restrict infection and stimulate immune responses [13]. In humans, the toll-like receptor family comprises ten identified members (TLR1 through TLR10) [14,15]. Subcellular localization differs across this family: TLR1, 2, 4, 5, 6, and 10 reside on the plasma membrane, whereas TLR3, 7, 8, and 9 are confined to endosomal/endoplasmic reticulum compartments [16]. Recent research has indicated that SARS-CoV-2 stimulates the innate immune system via TLRs and boosts TLR expression, resulting in the elimination of infection [17]. While the TLR response is to promote viral clearance, dysregulated TLR responses may direct continual inflammation and tissue destruction [18]. For instance, overactivation of TLR pathways upregulates a cytokine storm and exacerbates the severity of COVID-19 [6,19].

Among several TLRs, TLR8 recognizes single-stranded RNA and induces inflammatory responses via TLR8-dependent signaling during COVID-19 [20]. COVID-19 showed a different response according to sex; males showed more vulnerability and higher mortality compared to females [21]. Thus, several studies have suggested that immune-related genes located on the X chromosome are important factors in the pathomechanism of COVID-19, and the *TLR8* gene is noteworthy because it is located on the X chromosome [21,22,23]. Likewise, TLR8 expression was dramatically increased in patients with severe COVID-19 [24,25]. These results suggest a strong association between the *TLR8* gene and the pathomechanism of COVID-19.

Single-nucleotide polymorphisms (SNPs) are variations in DNA sequences that can alter gene transcription and protein function, driving variance across species and human beings [26,27,28]. Since SNP markers may be located across the genome and may be stably passed to progeny, they are employed in genetic and genome-wide association research [29,30]. Numerous SNPs are required to analyze population differences, especially in the case of pandemic diseases such as COVID-19 [31,32,33,34], as they provide information on individuals’ possible responses to certain treatments and susceptibilities to environmental infection [35]. It has been demonstrated that *TLR3* rs3775290, *TLR4* Asp299Gly and Thr399Ile, *TLR7* rs179008, and *TLR9* polymorphisms were correlated with the prognosis and susceptibility to SARS-CoV-2 infection [7,36,37]. Alhabibi et al. 2023 showed that *TLR2* rs5743708 and *TLR9* rs5743836 variants have been significantly associated with the severity of COVID-19 infection [38].

The presence of SNPs in important regulatory regions, such as the promoter region, may explain variances in cytokine production among people [39]. SNPs within TLR genes may reduce individuals’ ability to respond appropriately to TLR ligands, altering susceptibility to infectious diseases. Since SNPs in the *TLR8* gene may correlate with TLR8 expression levels, the association between regulatory genetic polymorphisms in *TLR8* and COVID-19 susceptibility warrants exploration.

This study set out to examine whether polymorphic variants in the proximal promoter of *TLR8* are associated with COVID-19 case status. To this end, we applied amplicon-based sequencing to characterize *TLR8* genotypes and allele distributions among Korean COVID-19 patients and controls, analyzing males and females separately.

## 2. Materials and Methods

### 2.1. Study Participants

This study enrolled 364 participants overall, divided into a COVID-19 patient group (*n* = 191) and a control group (*n* = 173). Patients were identified among individuals admitted to the isolation ward of Jeonbuk National University Hospital (Jeonju-si, Republic of Korea), whereas controls were unrelated, disease-free volunteers obtained through the Korea Biobank Network, with a mean age of 58.7 ± 14.2 years. Eligibility required that participants be male or female, have SARS-CoV-2 infection confirmed via real-time PCR, and self-identify as ethnically Korean; individuals with documented HIV, hepatitis B/C, or chronic pulmonary conditions were excluded from participation. Among the 191 patients (90 male, 101 female; mean age 56.6 ± 15.8 years), 23 had received at least one COVID-19 vaccine dose: 10 with AstraZeneca, 1 with the Janssen vaccine, and 12 with Pfizer-BioNTech (6 single-dose, 6 two-dose recipients). The control cohort included 80 males and 93 females, with the age distribution noted above.

### 2.2. Ethical Statements

All participants provided written informed consent before enrollment. This study adhered to the ethical principles of the Declaration of Helsinki and received approval from the Institutional Ethics Committee of Jeonbuk National University Hospital (approval code 2020-02-050-067; approval date, 19 October 2023).

### 2.3. Genomic DNA Extraction

Genomic DNA was extracted from 200 µL whole-blood specimens collected from each participant using a commercial blood DNA isolation kit (Qiagen, Valencia, CA, USA) following the manufacturer’s protocol. DNA yield and purity were quantified by A260/A280 absorbance measurements on a NanoDrop One spectrophotometer (Thermo Fisher Scientific, Waltham, MA, USA). Samples were then stored at −20 °C until further use.

### 2.4. Polymerase Chain Reaction

A single primer pair spanning the proximal promoter and first exon of *TLR8* (Gene ID: 51311) was designed using the Primer3Plus platform (Macrogen, Daejeon, Republic of Korea): forward, 5′-AGCCATTGACTCACTCGTTCA-3′; reverse, 5′-TGGGTCAGAAACCCCATATTC-3′. Each 25-µL PCR reaction contained 1 µL template DNA, 1 µL of each 10 µM primer, 2.5 µL 10× Taq polymerase buffer, 0.5 µL of 0.2 µM dNTPs, 5 µL 5× Band Helper, and 0.25 µL 10× Taq polymerase (BioFACT, Daejeon, Republic of Korea), following the manufacturer’s recommended cycling parameters. Thermocycling (C1000 Touch, Bio-Rad, Hercules, CA, USA) comprised an initial denaturation at 95 °C for 2 min, followed by 35 cycles of 95 °C/20 s, 58 °C/1 min, and 72 °C/1 min, with a final extension at 72 °C for 5 min. Amplicons were resolved on 1% agarose gels, stained with ethidium bromide, and visualized using a Gel Doc XR+ (Bio-Rad). Products were purified with a FavorPrep GEL/PCR kit (Favorgen Biotech, Ping Tung, Taiwan) and bidirectionally sequenced on an ABI 3730 platform (Applied Biosystems, Foster City, CA, USA) using BigDye Terminator v3.1 chemistry (Applied Biosystems) per the manufacturer’s instructions; sequencing was outsourced to Macrogen (Daejeon, Republic of Korea). To confirm genotyping reliability, 10% of samples were randomly re-sequenced independently, yielding 100% concordance with initial calls. The overall call rate reached 100% across all subjects and SNPs.

For genotyping analysis, PCR amplification followed by Sanger DNA sequencing using the chain-termination method (PCR-sequencing) was used to identify SNPs. Finch TV 1.4.0 software (Geospiza Corp., Seattle, WA, USA) was used to analyze the sequencing results [40]. The genotypes and alleles of *TLR8* polymorphisms were analyzed in patients and controls to investigate the association between *TLR8* polymorphisms and COVID-19 case status.

### 2.5. Statistical Analysis

Statistical analyses were performed using SPSS version 25.0 (IBM Corp., Armonk, NY, USA). Results were presented as frequencies (percentages) for qualitative variables (genotype, allele, and haplotype distributions) and as mean ± standard deviation (SD) for the quantitative variable age, and compared between groups using Student’s *t*-test. The chi-square (χ^2^) test or Fisher’s exact test was used to compare genotype and allele frequencies between the control group and COVID-19 patients. The odds ratio (OR) and 95% confidence interval (CI) for each group were assessed using logistic regression. Age adjustment was not performed because age was statistically balanced between cases and controls overall and within each sex-stratified group. However, residual confounding cannot be completely excluded, as acknowledged in the limitations section. To address multiple comparisons, the Benjamini–Hochberg false discovery rate (FDR) procedure was applied to the primary analysis (allele frequency comparisons of 4 SNPs in male subjects). An FDR threshold of *q* < 0.05 was used to determine significance. HWE was determined by testing the genotype frequencies at each SNP. Because the *TLR8* gene is located on the X chromosome, we tested for HWE only in females. We analyzed LD and haplotypes using Haploview version 4.2 (Broad Institute, Cambridge, MA, USA) based on the observed frequencies of four SNPs. For female subjects, five genetic models were tested for each SNP: the allele model (comparing minor and major allele frequencies), the codominant model (evaluating all three genotypes independently: homozygous major, heterozygous, and homozygous minor), the dominant model (comparing homozygous major versus heterozygous plus homozygous minor), the recessive model (comparing homozygous major and heterozygous group versus homozygous minor), and the overdominant model (comparing homozygous major and homozygous minor versus heterozygous). In contrast, for male subjects, only the allele model was used, as *TLR8* is X-linked and hemizygous males carry a single allele, rendering genotype-based models that assume two alleles per individual inapplicable. The sample size was calculated by G*Power (version 3.1.9.2; Germany). The calculation was based on a chi-square test of association in a 2 × 2 contingency table (allele × disease status) under the following parameters: a small-to-medium effect size of w = 0.15 [41], a two-sided significance level of α = 0.05, a statistical power of 80% (1−β = 0.80), one degree of freedom (df = 1), and an approximate allocation ratio of 1:1 (cases to controls). Under these assumptions, the minimum required total sample size was 349 participants. Our enrolled sample of 364 individuals (191 cases, 173 controls) exceeds this threshold. For individual tests, statistical significance was defined as *p* < 0.05. For the primary male allele comparisons, significance was further evaluated after FDR correction at *q* < 0.05.

## 3. Results

### 3.1. Patients and Samples

Table 1 summarizes specific information regarding the study population. There was no significant difference in age between the COVID-19 patients and the control group (*p* = 0.194).

### 3.2. The Location and Genotyping of SNPs

**Figure 1:**
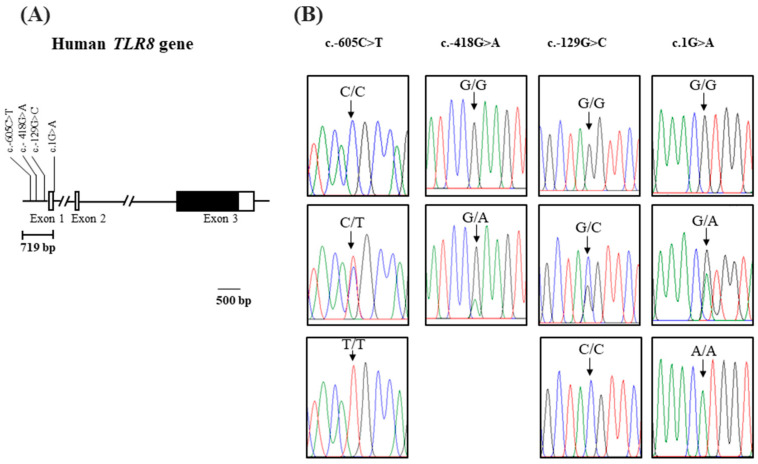
(**A**) Gene map and polymorphisms identified in the human toll-like receptor 8 gene (*TLR8*). The sequenced region is shown as horizontal bars (719 bp). The locations of single-nucleotide polymorphisms (SNPs) identified in this study are shown by arrows. A shaded block represents the open reading frame (ORF) in exon 3. (**B**) Electropherograms of the four SNPsidentified are shown: c.-605C>T, c.-418G>A, c.-129G>C, and c.1G>A. The upper/lower panels show homozygous genotypes; the middle panel shows heterozygous genotypes. Using an ABI 3730 automated sequencer, four colors show individual bases of a DNA sequence (blue: cytosine, red: thymine, black: guanine, and green: adenine).

*TLR8* genotype and allele distributions were determined via amplicon sequencing across the full cohort (173 controls, 191 patients). Alignment of the resulting sequences confirmed a match to the human *TLR8* reference sequence deposited in GenBank under Gene ID 51311. We uncovered a total of four SNPs, including c.-605C>T (rs5741883), c.-418G>A (rs186566524), c.-129G>C (rs3764879), and c.1G>A (rs3764880) (Figure 1A,B). Genotypic distributions of the 4 SNPs were consistent with HWE (*p* > 0.05). Because the *TLR8* gene is located on the X chromosome, only female subjects were included in the HWE test (Table 2). Our study also revealed that minor allele frequency (MAF) was similar to that reported in the public database of the Korean population (Table 2).

### 3.3. Associations Between COVID-19 Case Status and Genotype, Allele, and Haplotype Frequencies of TLR8 Polymorphisms

To address the study objective, allele and genotype frequencies were compared between female COVID-19 patients and female controls using multiple genetic models (Table 3). The analysis revealed no statistically significant association with COVID-19 case status under any genetic model (*p* > 0.05).

In males, the C allele of *TLR8* rs3764879 was present at frequencies of 24% and 10% in the control and patient groups, respectively. The difference was statistically significant and remained significant after FDR correction (OR 0.35; 95% CI 0.15–0.8; *p* = 0.018; *q* = 0.036). For *TLR8* rs3764880, the A allele had frequencies of 24% and 9% in the control and patient groups, respectively. The difference was statistically significant and remained significant after FDR correction (OR 0.3; 95% CI 0.12–0.7; *p* = 0.01; *q* = 0.036) (Table 4). Table 4 presents the full Benjamini–Hochberg FDR-adjusted q-values for all four SNPs, indicating that only rs3764879 and rs3764880 remained significant after multiple-comparison correction. Because there are only small numbers of male minor-allele carriers, these estimates should be interpreted with caution and confirmed in larger independent cohorts.

In addition, we performed haplotype analysis of four polymorphisms in the TLR8 gene in females using Haploview 4.2. The SNPs in the *TLR8* gene revealed three main haplotypes with no significant differences in COVID-19 case status among Korean females (*p* > 0.05) (Table 5).

Furthermore, LD was analyzed between the identified SNPs of the *TLR8* gene with *r2* values. In females, the *TLR8* rs3764879 showed a strong LD with rs3764880 in both the control and patient groups (*r2* = 0.805 and 0.914, Table 6).

## 4. Discussion

While some patients with COVID-19 demonstrate spontaneous resolution, the factors influencing the features of their immune responses remain mostly unidentified [42]. Host factors, including age, sex, and genetic factors, impact the spontaneous consequence after SARS-CoV-2 infection, the progression to fatality, or a response to treatment [43,44]. The *TLR8* gene is critical in the immunological response to SARS-CoV-2. Therefore, TLR7/8 agonists have been suggested as a therapeutic approach for COVID-19 treatment [20,45]. Male sex has been recognized as a potential risk factor for hospitalization and mortality following SARS-CoV-2 infection. On the other hand, females often have a stronger immunological response than males, and X chromosomal genes might explain this tendency [46].

Given the recognized contribution of TLR8 to innate antiviral defense against SARS-CoV-2, we sought to determine whether *TLR8* polymorphisms are associated with COVID-19 case status in a Korean cohort. Amplicon sequencing from 173 controls and 191 patients identified four candidate regulatory SNPs in the promoter and first exon of TLR8. Previous studies have suggested that SNPs in *TLR8* may exhibit sex-specific effects in genetic association studies [47]. Consistent with these findings, our results revealed a sex-specific association between *TLR8* rs3764879 and rs3764880 in males, but not in females. It has been shown that *TLR8* rs3764879 and/or rs5741883 are associated with tuberculosis (TB) infection [48], allergic rhinitis [49], Chikungunya virus (CHIKV) infection [50], and systemic lupus erythematosus [51]. Here, we observed that the allele frequencies of *TLR8* rs3764879 differed significantly between male COVID-19 patients and the control group, suggesting an association with COVID-19 case status, rather than a confirmed causal role in infection risk. This result is inconsistent with a recent study by Bagci et al. (2023), which reported no association between the *TLR8* rs3764879 SNP and COVID-19 [9]. This discrepancy may be attributed to the genetic differences among ethnic groups [52]. Although our control group was not formally matched to cases at the individual level, the two groups had comparable age and sex distributions, and all association analyses were stratified by sex, the primary demographic variable relevant to X-linked gene analysis.

Several studies have investigated the *TLR8* rs3764880 polymorphism and its association with susceptibility to TB infection across different ethnicities [53]. For instance, in the Chinese population, the G allele of *TLR8* rs3764880 was associated with protection against TB [54]. In the Turkish population, the A allele of the same polymorphism was associated with susceptibility to pulmonary TB [55]. The *TLR8* rs3764880 SNP was also found to be associated with protection against the development of acquired immunodeficiency syndrome in both males and females [56]. Our findings indicated that the A allele frequency is significantly higher in the control group than in male COVID-19 patients. Given the strong LD between rs3764879 and rs3764880 (*r*^2^ = 0.805–0.914, Table 6), these two SNPs likely tag the same haplotype and should be interpreted as a single genetic signal rather than two independent associations. This result is consistent with findings in Italian, Russian, and Turkish populations [9,57,58].

No prior study has examined a potential link between rs186566524 and COVID-19. Our findings indicate that this variant shows no detectable association with COVID-19 case status in this Korean cohort. It has been indicated that males have higher degrees of mortality and clinical problems from COVID-19 compared to females [59]. Among TLRs, Fallerini and his colleagues showed an association between *TLR7* variants (rs189681811, rs147244662, rs149314023, rs200146658, and rs5743781) and COVID-19 in males [60]. Taha et al. identified a link between the *TLR4* (Asp299Gly and Thr399Ile) minor alleles 299Gly (G) and 399Ile (T) and COVID-19 severity in a male-dominated sample [36].

We also analyzed the haplotype frequencies of the *TLR8* gene in female subjects, comparing the control group and COVID-19 patients, and found no significant differences in haplotype frequencies according to COVID-19 case status (Table 5). In contrast, the individual SNP analysis showed male-specific associations of rs3764879 and rs3764880 with COVID-19 case status (Table 4). A strong genetic linkage was identified between *TLR8* rs3764879 and rs3764880 in both the control and patient groups. Notably, other SNPs exhibited weak LD values. TLRs respond to pathogens by stimulating acquired immunity, including the release of proinflammatory cytokines [61]. Among TLRs, interactions between COVID-19 and TLR variants have been explained, mainly for TLR3, TLR4, and TLR7. It has been reported that the TLR2-9 pool has facilitated the clearance of SARS-CoV-2 [62]. *TLR3* rs3775290 and *TLR7* rs179008 SNPs were indicated to be substantially linked with an extremely high risk of COVID-19 pneumonia, but not with disease outcome [7]. The GG homozygote at the *TLR7* rs3853839 SNP was significantly more common among COVID-19 patients (*n* = 150) than among controls (*n* = 135). As a result, these SNPs have been associated with SARS-CoV-2 infection, cytokine storms, and higher patient mortality rates [63]. On the other hand, Zayed et al. showed that the *TLR7* (rs864058) polymorphism is not associated with SARS-CoV-2 infection in the Korean population [64].

In the post-pandemic genetics era, large-scale GWAS studies have identified strong autosomal susceptibility loci for COVID-19. However, X-linked immune genes such as *TLR7* and *TLR8* remain underexplored because standard GWAS methods struggle to analyze hemizygous loci. Our research adds to this knowledge by offering population-specific, sex-stratified data from a non-European cohort.

Our study design identified associations between *TLR8* genetic variants and confirmed COVID-19 case status (Table 4). Although these results suggested a potential role in the risk of SARS-CoV-2 infection, we cannot rule out that the observed associations reflect differences in disease progression or clinical symptoms rather than direct risk of infection. This study has potential limitations that should be acknowledged. First, the relatively small sample size, limited to Jeonju, South Korea, may introduce bias into participant selection and data analysis. The small number of minor allele carriers among males results in wide confidence intervals, so the reported associations are preliminary and require validation in larger, independent cohorts. Second, age-adjusted logistic regression was not performed. Although age was statistically balanced between groups, the absence of a significant age difference does not completely exclude residual confounding. Residual confounding could influence the observed associations, and future studies with larger sample sizes should address this. Third, the risk of batch effects, arising from using pre-existing biobank samples as controls processed at a different time from the patient samples, cannot be entirely ruled out, despite our efforts to maintain consistent protocols. Fourth, the control participants from the biobank were not verified as SARS-CoV-2-negative, and their infection history, immune status, vaccination status, and COVID-19-related symptoms remain unknown. As a result, some controls could have had previously unrecognized or asymptomatic SARS-CoV-2 infections. Undetected asymptomatic infection among controls could bias the observed odds ratios in either direction, thereby limiting causal interpretation. This limits the interpretation of the observed associations, as they might not reflect true susceptibility but could instead be influenced by differences in infection detection, disease symptoms, or clinical course. Finally, while the genetic homogeneity of our Korean population reduces the risk of population stratification confounding our results, it also limits the applicability of our findings. Functional validation, including quantification of TLR8 expression, eQTL analysis, and cytokine profiling, was beyond the scope of the present association study and is a defined priority for follow-up investigation. Future replication in diverse, multi-ethnic cohorts is necessary to confirm the broader relevance of these *TLR8* variants in COVID-19 susceptibility.

## 5. Conclusions

In conclusion, this study demonstrated a significant risk association between a *TLR8* haplotype in the proximal promoter–exon 1 region, tagged by the correlated SNPs rs3764879 and rs3764880 (*r*^2^ > 0.8), and COVID-19 case status, with sex-dependent effects in the Korean population. We believe that, in developing future individual risk profiles for SARS-CoV-2 infection, patients’ sex should be taken into account. Further validation in larger independent cohorts is required to confirm these findings.

## Figures and Tables

**Table 1:** Detailed information on the study population.

Characteristics	Controls	COVID-19 Patients	*p*-Value
Number, *n*	173	191	
Sex			
Male, *n* (%)	80 (46)	90 (47)	
Female, *n* (%)	93 (54)	101 (53)	
Age (year ± SD), overall	58.7 ± 14.2	56.6 ± 15.8	0.194
Age (year ± SD), male	57.5 ± 12.9	58.9 ± 14.6	0.520
Age (year ± SD), female	59.7 ± 14.9	54.8 ± 16.5	0.086

**Table 2:** SNPs of *TLR8* and HWE in this study.

SNPs	Location	Nucleotide	Groups	HWE	MAF (Korean)
rs5741883	X:12906102 (GRCh38)	C>T	Control (*n* = 93)	0.6	T = 0.02
			Patients (*n* = 101)	0.747	
rs186566524	X:12906289 (GRCh38)	G>A	Control (*n* = 93)	0.639	A = 0.01
			Patients (*n* = 101)	0.874	
rs3764879	X:12906578 (GRCh38)	G>C	Control (*n* = 93)	0.475	C = 0.19
			Patients (*n* = 101)	0.274	
rs3764880	X:12906707 (GRCh38)	G>A	Control (*n* = 93)	0.295	A = 0.19
			Patients (*n* = 101)	0.06	

Abbreviations: SNPs, single-nucleotide polymorphisms; HWE, Hardy–Weinberg equilibrium; MAF, minor allele frequency.

**Table 3:** Association analysis between *TLR8* polymorphisms and COVID-19 case status in female individuals using different genetic models.

SNPs	Models	Genotype	Controls, *n* (%)	Patients, *n* (%)	OR (95% CI)	*p*-Value
**rs5741883**	Allele	C T	180 (97) 6 (3)	192 (95) 10 (5)	Reference 1.5 (0.5–4.3)	0.396
	Codominant model	CC CT TT	87 (93.5) 6 (6.5) 0 (0.0)	91 (90) 10 (10) 0 (0.0)	Reference 1.6 (0.5–4.5)	0.386 NA
	Dominant model	CC CT+TT	87 (93.5) 6 (6.5)	91 (90) 10 (10)	Reference 1.6 (0.5–4.5)	0.386
	Recessive model	CC+CT TT	93 (100) 0 (0.0)	101 (100) 0 (0.0)	Reference	NA
	Overdominant model	CC+TT CT	87 (93.5) 6 (6.5)	91 (90) 10 (10)	Reference 1.6 (0.5–4.5)	0.386
**rs186566524**	Allele	G A	183 (98) 3 (2)	193 (95.5) 9 (4.5)	Reference 2.8 (0.7–10.6)	0.121
	Codominant model	GG GA AA	90 (97) 3 (3) 0 (0.0)	92 (91) 9 (9) 0 (0.0)	Reference 2.9 (0.7–11.2)	0.114 NA
	Dominant model	GG GA+AA	90 (96.8) 3 (3.2)	92 (91) 9 (9)	Reference 2.9 (0.7–11.2)	0.114
	Recessive model	GG+GA AA	93 (100) 0 (0.0)	101 (100) 0 (0.0)	Reference	NA
	Overdominant model	GG+AA GA	90 (97) 3 (3)	92 (91) 9 (9)	Reference 2.9 (0.7–11.2)	0.114
**rs3764879**	Allele	G C	149 (80) 37 (20)	161 (80) 41 (20)	Reference 1.0 (0.6–1.6)	0.920
	Codominant model	GG GC CC	58 (62) 33 (36) 2 (2)	63 (62) 35 (35) 3 (3)	Reference 1.0 (0.5–1.7) 1.3 (0.2–8.5)	0.937 0.728
	Dominant model	GG GC+CC	58 (62.4) 35 (37.6)	63 (62.4) 38 (37.6)	Reference 1.0 (0.5- 1.7)	0.998
	Recessive model	GG+GC CC	91 (98) 2 (2)	98 (97) 3 (3)	Reference 1.3 (0.2–8.5)	0.720
	Overdominant model	GG+CC GC	60 (64.5) 33 (35.5)	66 (65.3) 35 (34.7)	Reference 0.9 (0.5–1.7)	0.903
**rs3764880**	Allele	G A	155 (83) 31 (17)	158 (78) 44 (22)	Reference 1.3 (0.8–2.3)	0.203
	Codominant model	GG GA AA	62 (67) 31 (33) 0 (0.0)	60 (60) 38 (38) 3 (3)	Reference 1.2 (0.7–2.2)	0.434 0.193
	Dominant model	GG GA+AA	62 (66.6) 31 (33.4)	60 (59.4) 41 (40.6)	Reference 1.3 (0.7–2.4)	0.296
	Recessive model	GG+GA AA	93 (100) 0 (0.0)	98 (97) 3 (3)	Reference	0.212
	Overdominant model	GG+AA GA	62 (66.6) 31 (33.4)	63 (62.4) 38 (37.6)	Reference 1.2 (0.6–2.1)	0.533

Notes: *p*-value > 0.05, non-significant. Abbreviations: OR, odds ratio; CI, confidence interval. NA, not applicable.

**Table 4:** Association analysis between *TLR8* SNPs and COVID-19 case status in male individuals.

SNPs	Controls, *n* (%)	Patients, *n* (%)	OR (95% CI)	*p*-Value	*q*-Value
**rs5741883**					
C T	79 (99.0) 1 (1.0)	90 (100) 0 (0.0)	Reference	0.454	0.454
**rs186566524**					
G A	79 (99.0) 1 (1.0)	87 (96.6) 3 (3.4)	Reference 2.7 (0.27–26.7)	0.389	0.454
**rs3764879**					
G C	61 (76.0) 19 (24.0)	81 (90.0) 9 (10.0)	Reference 0.35 (0.15–0.8)	0.018	0.036
**rs3764880**					
G A	61 (76.0) 19 (24.0)	82 (91.0) 8 (9.0)	Reference 0.3 (0.12–0.7)	0.01	0.036

Notes: *p*-value > 0.05 non-significant, *p*-value < 0.05 significant. Abbreviations: OR, odds ratio; CI, confidence interval. Benjamini–Hochberg FDR correction was applied across all 4 SNP comparisons; rs3764879 and rs3764880 remained significant at *q* < 0.05, while rs5741883 and rs186566524 are not significant.

**Table 5:** Comparison of haplotype frequencies of *TLR8* polymorphisms associated with COVID-19 case status in Korean females.

Haplotypes	Frequency		*p*-Value
	Control (*n* = 186)	Patients (*n* = 202)	
CGGG	150 (0.806)	147 (0.728)	0.4679
CGCA	23 (0.124)	32 (0.158)	1.0
TGCA	5 (0.027)	7 (0.035)	0.6780
Other	8 (0.043)	16 (0.079)	-

*n* = total number of haplotypes. Frequency = proportion of each haplotype among total haplotypes within each group. Other represents pooled rare haplotypes, each present at <5% frequency, combined for statistical comparison due to low individual counts.

**Table 6:** Linkage disequilibrium (LD) among single-nucleotide polymorphisms (SNPs) of the human toll-like receptor 8 gene (*TLR8*) in a Korean population.

Female	rs5741883	rs186566524	rs3764879	rs3764880
rs5741883	-	0.008	0.137	0.132
rs186566524	0.042	-	0.00	0.00
rs3764879	0.134	0.066	-	**0.914**
rs3764880	0.167	0.082	**0.805**	-

Bold text indicates strong LD (*r2* > 0.3). The above diagonal indicates the LD value in COVID-19 patients. The below diagonal indicates the LD value in controls.

## Data Availability

The data presented in this study are available on request from the corresponding authors due to ethical reasons.
